# Enhanced Biosynthesis of 2-Deoxy-*scyllo*-inosose in Metabolically Engineered *Bacillus subtilis* Recombinants

**DOI:** 10.3389/fmicb.2018.02333

**Published:** 2018-09-27

**Authors:** Joo Hyun Lim, Hyun Ha Hwang, Na Joon Lee, Jae Woo Lee, Eun Gyo Seo, Hye Bin Son, Hye Ji Kim, Yeo Joon Yoon, Je Won Park

**Affiliations:** ^1^Department of Integrated Biomedical and Life Sciences, Graduate School, Korea University, Seoul, South Korea; ^2^Department of Chemistry and Nanoscience, Ewha Womans University, Seoul, South Korea; ^3^School of Biosystem and Biomedical Science, Korea University, Seoul, South Korea

**Keywords:** 2-deoxy-*scyllo*-inosose, *Bacillus subtilis*, metabolic engineering, artificial gene, 2-deoxy-*scyllo*-inosose synthase

## Abstract

2-Deoxy-*scyllo*-inosose (DOI) has been a valuable starting natural product for the manufacture of pharmaceuticals or chemical engineering resources such as pyranose catechol. DOI synthase, which uses glucose-6-phosphate (Glc6P) as a substrate for DOI biosynthesis, is indispensably involved in the initial stage of the biosynthesis of 2-deoxystreptamine-containing aminoglycoside antibiotics including butirosin, gentamicin, kanamycin, and tobramycin. A number of metabolically engineered recombinant strains of *Bacillus subtilis* were constructed here; either one or both genes *pgi* and *pgcA* that encode Glc6p isomerase and phosphoglucomutase, respectively, was (or were) disrupted in the sugar metabolic pathway of the host. After that, three different DOI synthase–encoding genes, which were artificially synthesized according to the codon preference of the *B. subtilis* host, were separately introduced into the engineered recombinants. The expression of a natural *btrC* gene, encoding DOI synthase in butirosin-producing *B. circulans*, in the heterologous host *B. subtilis* (BSDOI-2) generated approximately 2.3 g/L DOI, whereas expression of an artificially codon-optimized *tobC* gene, derived from tobramycin-producing *Streptomyces tenebrarius*, into the recombinant of *B. subtilis* (BSDOI-15) in which both genes *pgi* and *pgcA* are disrupted significantly enhanced the DOI titer: up to 37.2 g/L. Fed-batch fermentation by the BSDOI-15 recombinant using glycerol and glucose as a dual carbon source yielded the highest DOI titer (38.0 g/L). The development of engineered microbial cell factories empowered through convergence of metabolic engineering and synthetic biology should enable mass production of DOI. Thus, strain BSDOI-15 will surely be a useful contributor to the industrial manufacturing of various kinds of DOI-based pharmaceuticals and fine chemicals.

## Introduction

Pyranose compounds have been produced in the traditional petrochemical sector from petroleum as a raw material. Most of these aromatic compounds including catechol and benzenoids are still being made from petroleum (Hansen and Frost, 2002; Baire et al., 2013), but because of limited petroleum reserves and worldwide regulations on carbon dioxide emissions, the development of environment-friendly and sustainable production processes using biomass (e.g., a fermentation process) is in demand.

2-Deoxy-*scyllo*-inosose (DOI) synthase, which uses Glc6P as a substrate when catalyzing the synthesis of pyranose compound DOI was first discovered as BtrC in *Bacillus circulans* that produces 2-deoxystreptamine-containing aminoglycoside butirosins (Kudo et al., 1999). This enzyme participates in the biosynthetic steps necessary for the core 2-deoxystreptamine scaffold (Llewellyn and Spencer, 2006; Park et al., 2013), and its catalytic product DOI has been broadly utilized as a starting material or a precursor of agrochemicals and pharmaceuticals (Hansen and Frost, 2002). Chemical synthesis of this DOI requires multistep reactions and hazardous and expensive metals, whereas the biosynthesis of DOI by DOI synthase allows for efficient synthesis in a single process. A method for producing DOI in a single enzymatic reaction was established (Takagi et al., 2006) in which a recombinant DOI synthase expressed in *Escherichia coli* is mixed with Glc6P. In addition, there was a report about a two-step enzymatic reaction that involves hexokinase and DOI synthase acting on D-glucose (Kakinuma et al., 2000). Furthermore, it was also reported that by the concentration of the enzymatic reactants followed by a reaction with hydrogen iodide under mild acidic conditions, DOI can be converted even to catechol (Suzuki et al., 2013).

Meanwhile, there was a publication concerning the biosynthesis of DOI via microbial fermentation of biomass-derived glucose by a recombinant strain of *E. coli*, into which a heterologous DOI synthase-encoding gene was introduced (Kogure et al., 2007). Accordingly, along with glucose, the rare and expensive sugar alcohol mannitol was also required as an extra carbon source for the support of microbial proliferation and growth of *E. coli*. In other words, having DOI synthase expressed in a wild-type strain of *E. coli* alone resulted in low productivity in terms of DOI, but high production of DOI (29.5 g/L), as reported, was achieved by simultaneous disruption of three genes essential for the primary metabolic pathway of *E. coli*: phosphoglucose isomerase (*pgi*), glucose 6-phosphate-1-dehydrogenase (*zwf*), and phosphoglucomutase (*pgm*). With all the metabolic pathways via which glucose can enter glycolysis eventually being blocked, mannitol that can be used in alternate glycolysis should be requisite for *E. coli* growth. In addition, a method for producing DOI from plant-derived ingredients, including sucrose as the less expensive carbon source than glucose, was reported (Tokuda et al., 2011). That is, a recombinant strain of *E. coli* that harbors both a sucrose-6-phosphate hydrolase (CscA) gene and a DOI synthase (BtrC) gene was created, through which a cost-effective and scalable fermentation process that produces DOI from sucrose as the major ingredient of molasses, was next developed. Moreover, a novel DOI synthase that shows relatively higher heat resistance and pH stability than the existing DOI synthases was also discovered (Konishi and Imazu, 2010). DOI synthase derived from the *Streptoalloteichus hindustanus* JCM3268 strain was also discovered (Hirayama et al., 2006). Most recently, there was a report on overexpression of the above *btrC* gene in the photoautotrophic cyanobacterium *Synechococcus elongatus*, resulting in 400 mg/L DOI photosynthesis without any need for a carbon source for the recombinant (Watanabe et al., 2018).

**FIGURE 1:**
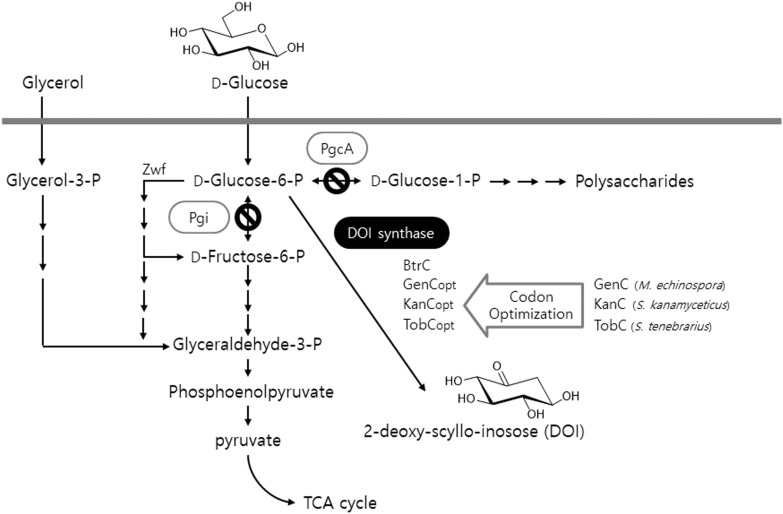
An illustration of the production of DOI by the recombinant strains of *B. subtilis* via both engineering of the glycolytic metabolic pathway in the host and expression of codon-optimized artificial DOI synthases. TCA: tricarboxylic acid.

As mentioned above, DOI synthase is a crucial enzyme that is involved in the beginning of the biosynthesis of 2-deoxystreptamine-containing aminoglycoside antibiotics. Therefore, the gene encoding DOI synthase must be present typically within a number of biosynthetic gene clusters essential for the biosynthesis of relevant antibiotics such as gentamicin, kanamycin, and tobramycin: *genC* originating from gentamicin-producing *Micromonospora echinospora, kanC* from kanamycin-producing *Streptomyces kanamyceticus*, and *tobC* from tobramycin-producing *Streptomyces tenebrarius* (Park et al., 2013). Herein, three codon-optimized artificial genes (*genC_opt_, kanC_opt_*, and *tobC_opt_*) were synthesized from the previously described *genC, kanC*, and *tobC* sequence templates (GenBank accession numbers AJ628149, AJ628422, and AJ810851, respectively) according to the codon usage preference of *B. subtilis* (**Figure 1**). Next, the glycolytic metabolic pathway in the heterologous host *B. subtilis* into which the above-mentioned artificial genes were being separately introduced was engineered; either one or both genes *pgi* and *pgcA*, which encode Glc6P isomerase and phosphoglucomutase, respectively, was (or were) disrupted in the primary metabolic pathway of the host (**Figure 1**). On the other hand, a gene *zwf* encodes Glc6P-1-dehydrogenase which plays its role in the branched routes from Glc6P as an initiator of oxidative pentose phosphate pathway (**Figure 1**). Based on the previous report (Zamboni et al., 2004), the *zwf* knockout mutant showed about 1.5-folds reduced growth rate compared with the wild-type strain. However, in case of other two genes (*pgi* and *pgcA*), there has been no report for the negative effect on the host growth. Therefore, in this study, we constructed Δpgi and ΔpgiΔpgcA knockout mutants, in which the *zwf* gene is still intact. The present study involves the design and construction of a number of metabolically engineered recombinant strains of *B. subtilis* into which several natural and artificial DOI synthase–encoding genes were introduced. This approach should ultimately provide a microbial cell factory platform that can produce DOI with a high titer and productivity, as compared to the existing technology.

## Materials and Methods

### Construction of Bacterial Strains and Plasmids

Strain *B. subtilis* 168 (genotype: *trpC2*) served as a negative control (Belda et al., 2013). A gene-targeting method that does not require selection markers (Fabret et al., 2002) was employed for the construction of recombinant strains of *B. subtilis*, in which either one or both *pgi* (Glc6P isomerase gene) and *pgcA* (phosphoglucomutase gene) involved in the glycolysis metabolic pathway (KEGG pathway ID bsu00010) was (or were) disrupted in the genome of the host. After transformation of integration plasmid pCU-pgiFB (phenotype: Cm^R^ [chloramphenicol resistance]) into *B. subtilis* strain 168, it was inserted into the *B. subtilis* genome via the first single-crossover recombination. The transformed recombinant Δpgi strain (genotype: *trpC2*Δ*pgi*, phenotype: Cm^R^) was selected on a Luria-Bertani (LB; BD Biosciences, Sparks, MD, United States) solid plate medium supplemented with 5 μg/mL chloramphenicol (Sigma-Aldrich, St. Louis, MO, United States). After cultivation in the liquid LB medium for 18 h, the recombinants were counterselected on the minimal medium (MM) supplemented with 10 μM 5-fluorouracil (Sigma-Aldrich). Similarly, another integration plasmid, pCU-pgcAFB (phenotype: Cm^R^), was introduced into the recombinant Δpgi strain to obtain the recombinant ΔpgiΔpgcA strain (genotype: *trpC2*Δ*pgi*Δ*pgcA*, phenotype: Cm^R^) with both genes *pgi* and *pgcA* knocked out.

On the other hand, constitutive gene expression plasmid pHP13-P43 was constructed in the following manner. First, P_43_, one of the promoters originating from *B. subtilis*, was amplified by PCR using a forward primer, P43-F (5′GGTAAAGCTTGCGGCTTCCTTGTAGAGCTCAG3′, underlined is a HindIII restriction enzyme cleavage site) and reverse primer P43-B (5′CTCTCTGCAGCATGTGTACATTCCTCTC3′, underlined is a PstI site). P_43_ promoter has been routinely utilized for the expression of heterologous gene in *B. subtilis*, as it is strong constitutive promoter (Song et al., 2016). After cleavage with both HindIII and PstI, the PCR products were ligated to *B. subtilis* expression plasmid pHP13 (*Bacillus* Genetic Stock Center, Columbus, OH, United States) that was digested with the same restriction enzymes, thus generating pHP13-P43 (genotype: P_43_, phenotype: Cm^R^, Em^R^ [erythromycin resistance]).

The above-mentioned integration plasmid pCU-pgiFB was constructed in the following manner. The upper and lower DNA fragments of the *pgi* gene within the *B. subtilis* genome, pgi-F and pgi-B, respectively, were amplified from the *B. subtilis* 168 strain genome as a template with a pair of primers: D-pgi-FU/L (5′CTAAACATGAACTGACAATTGAGGAAG3′) and D-pgi-BU/L (5′GAAGAAATATACAAGGTATCCAAAAGTATATG3′). Then, after fusion of these two DNA fragments, fusion PCR was performed with the D-pgi-FsnU/L primer. The PCR products were cleaved with restriction enzymes SphI and KpnI, and pCU-pgiFB was generated by ligating the amplicons to pCU (phenotype: Cm^R^) that was digested with the same restriction enzymes. The other integration plasmid pCU-pgcAFB was produced by fusion PCR with equivalent primers (D-pgcA-FU/L: 5′TTAAGTTTATCGGTGAAAAGATTAAGGAATAC3′ and D-pgcA-BU/L: 5′AAAACCATATTCGTTAAAGAGATTGATGAG3′) and the same restriction enzyme sites.

The expression plasmid pHP13-P43-BtrC used for introducing *btrC*, a *B. circulans*-derived DOI synthase encoding gene, into *B. subtilis* strain 168 was assembled as follows. In other words, the genomic DNA of *B. circulans* NRRL B3312 was prepared as a template, and then subjected to PCR with forward primer BtrC-F (5′GTGGGTACCGAGGTTAAACATGACTAAAC3′, underlined is a KpnI site) and reverse primer BtrC-B (5′-CTCCTGCAGTTGTTATCGTGGATTAAATAATGG3′, underlined is a PstI site). After cleavage by both KpnI and PstI, the PCR products were ligated to expression plasmid pHP13-P43 that was digested with the same restriction enzymes, thereby yielding pHP13-P43-BtrC (genotype: P_43_-*btrC*, phenotype: Cm^R^, EM^R^).

To amplify the expression of DOI synthase–encoding genes derived from microbes other than *Bacillus* (*genC* [*Micromonospora echinospora* DSM 43036, GenBank accession number AJ628149], *kanC* [*Streptomyces kanamyceticus* DSM 40500, GenBank AJ628422], and *tobC* [*Streptomyces* sp. DSM 40477, GenBank AJ810851]), the codon-optimized gene fragments (*genC_opt_, kanC_opt_*, and *tobC_opt_*) were designed and artificially synthesized by CosmoGenetech (Seoul, Korea) according to the codon usage preference of the heterologous host *(B. subtilis)*. To all the codon-optimized gene fragments, we added a KpnI restriction site upstream of the ribosome-binding site and a PstI restriction site downstream of the termination codon, respectively. Three different artificial genes were treated with KpnI and PstI, and then ligated into plasmid pHP13-P43 that had been digested with the same restriction enzymes, thus generating pHP13-P43-GenC_opt_ (genotype: P_43_-*genC*_opt_, phenotype: Cm^R^, EM^R^), pHP13-P43-KanC_opt_ (genotype: P_43_-*kanC*_opt_), and pHP13-P43-TobC_opt_ (genotype: P_43_-*tobC*_opt_), respectively.

Gene manipulation was performed by standard techniques and transformation of both *E. coli* and *B. subtilis* was carried out by heat shock transformation and the natural competent transformation (Green and Sambrook, 2012). Antibiotics were added to the medium of recombinant strains at appropriate concentrations (kanamycin 5 μg/mL, ampicillin 100 μg/mL, and chloramphenicol 5 μg/mL; all from Sigma-Aldrich). All amplicons were routinely verified by sequencing.

### Shake-Flask Fermentation by the Recombinant Strains of *B. subtilis*

Unless specified otherwise, *E. coli* and recombinant *B. subtilis* strains were cultivated in the LB medium at 37°C. To examine the growth of the recombinant strains and their DOI productivity, 20 mL of the 2YTG liquid medium (1.6% tryptone, 1% yeast extract, 0.5% NaCl, 3% glucose, all as w/v) was placed into a 250 mL baffled Erlenmeyer flask. After that, each recombinant strain was inoculated and cultivated for 12 h under the conditions mentioned above. A slightly modified 2YTG medium (1.6% tryptone, 1% yeast extract, 0.5% NaCl, 3% glucose, 2% glycerol; w/v) which contains, as another carbon source, 2% of glycerol in addition to glucose was prepared for a fed-batch fermentation process. After inoculation into 250 mL of the shake-flask fermentation medium in a 1 L baffled Erlenmeyer flask, each recombinant was cultivated for up to 60 h at 37°C by reciprocal shaking at 200 rpm. Meanwhile, after 30 h of this shake-flask fermentation, 5 mL of a glucose solution (0.5 g/mL) that had been prepared under sterile conditions was added to the fed-batch culture to test whether the DOI productivity increases.

### Growth Curves of the Recombinant Strains and Analyses of Their DOI Titers

During the entire 60 h of fed-batch fermentation, an aliquot (2 mL) of fermentation broth was collected every 10 h to construct the growth curve in the following manner: 1 mL of serially diluted broth was placed into the UV-Vis spectrophotometer (Shimadzu, Japan) with absorbance measured at 680 nm. After refrigerated centrifugation of the same sample for 3 min at 5000 rpm (or ∼8000 × *g*), the precipitated cell debris were freeze-dried and then weighed. The linear curve of correlation between the dry cell weights and absorbance values was drawn, with which the dry cell weights of the recombinant strains of *B. subtilis* were determined during the fed-batch fermentation.

**FIGURE 2:**
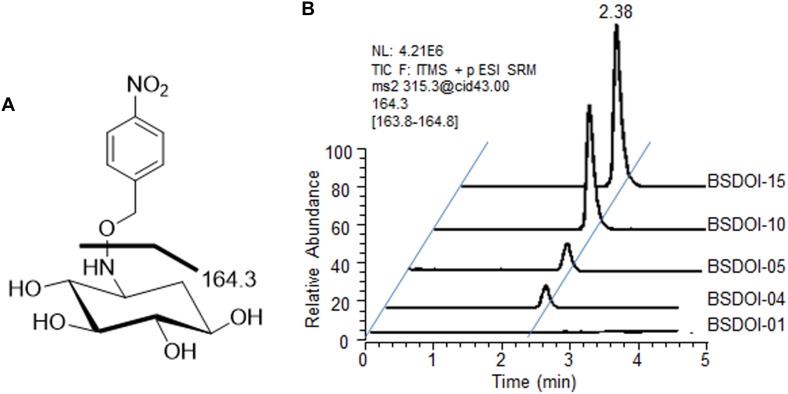
UPLC-ESI-MS/MS analysis of DOI produced by the recombinant strains of *B. subtilis*. **(A)** An ESI-MS/MS fragmentation pattern (*m/z* 315.3 > 164.3) of a DOI oxime derivative produced by the recombinants described herein. **(B)** A typical UPLC-ESI-MS/MS chromatogram for quantification of the DOI titer in the shake-flask fermentation broths of the recombinants.

On the other hand, quantification of DOI produced via the fed-batch fermentation by the recombinants was performed by means of a modified version of the procedures described in other reports (Yamauchi and Kakinuma, 1992; Kharel et al., 2004) as follows: 100 μL of the supernatant obtained by the above-mentioned refrigerated centrifugation was mixed with 300 μL of an aqueous methanol solvent (methanol/water at 1:2, v/v) together with 40 μL of *O*-(4-nitrobenzyl) hydroxylamine hydrochloride (Sigma-Aldrich), 35 mg/mL. The oxime adducts of DOI were prepared via the reaction for 30 min in a water bath set to 60°C (Kharel et al., 2004), and then the reactants were evaporated to dryness at room temperature by vacuum centrifugation. The DOI derivatives were reconstituted in 100 μL of methanol, and an aliquot (25 μL) was subjected to ultra high-performance liquid chromatography (UPLC) with electrospray ionization (ESI) and ion trap tandem mass spectrometry (MS/MS) analysis. The Spectra system P1000XR UPLC consists of a pump (Thermo Finnigan, San Jose, CA, United States) and a Spectra Series AS3000 autosampler (Thermo Finnigan, 20 μL loop). Isocratic chromatography was conducted on an Acquity CSH C_18_ (Waters, Milford, MA, United States) reversed-phase column at a flow rate of 120 μL/min of the mobile phase (acetonitrile/methanol/water/formic acid 1:1.5:8:0.002, v/v/v/v). The column effluent was directed to an LCQ ion trap mass spectrometer (Thermo Finnigan), operated in positive ion mode. The mass transition specific to DOI oxime adducts was *m/z* 315.3 > 164.3 (**Figure 2A**). Quantification of DOI produced during the fed-batch fermentation by each recombinant was carried out according to the calibration equation obtained from the correlation between the chromatographic peak areas and the concentration of the oxime adducts of an authentic DOI standard (GeneChem Inc., Daejeon, Korea). Each fermentation broth sample was also centrifuged, and then the supernatant of the sample was processed using two kinds of commercially available kits such as the D-glucose HK assay kit (Megazyme International Ltd., Wicklow, Ireland) and glycerol determination kit (Sigma-Aldrich) to determine the residual concentration of glucose and glycerol that had been added as carbon sources during fed-batch fermentation.

Growth curves and the DOI titer of recombinant strains of *B. subtilis*, together with the remaining glucose and glycerol content in the fed-batch fermentation process, were presented by averaging the results in quadruplicate.

## Results and Discussion

### Comparison of the DOI Titer Produced by the Recombinants That Express a Heterologous DOI Synthase

Among the recombinant *B. subtilis* strains that carried out 60 h of shake-flask fermentation as described before, DOI productivity of four recombinant strains that expressed DOI synthase solely without further metabolic pathway engineering was determined and compared with that of strain BSDOI-01 as a negative control (**Table 1**). When a *btrC* gene that encodes the DOI synthase from *B. circulans* was expressed in the *B. subtilis* host (BSDOI-02), an average of 2.3 g/L DOI was produced. A recent publication revealed that DOI production of 1.5 g/L can be achieved through expression of the identical *btrC* gene in *E. coli* as a host (Kogure et al., 2007). Meanwhile, when an artificial gene—*genC_opt_* synthesized on the basis of a previously described *genC* sequence template—was introduced into the host (BSDOI-03), DOI titer in the shake-flask fermentation broth remained at 0.8 g/L on average, showing a failure of the enhancement of DOI productivity through *genC_opt_* expression. In contrast, heterologous expression of *kanC_opt_* and *tobC_opt_*, both of which originate from the *Streptomyces* genus, in *B. subtilis* hosts (BSDOI-04 and BSDOI-05) increased the DOI titer up to 3.4 and 3.6 g/L, respectively (**Figure 2B**). In particular, the GC contents of two codon-optimized artificial genes *kanC_opt_* and *tobC_opt_* (from *Streptomyces*) are 52 and 59% respectively, whereas that of *genC_opt_* (from *Micromonospora*) is 68% even being synthesized according to the codon-usage preference in *B. subtilis* host, suggesting the negative effect of high GC-content onto the gene expression in heterologous host. Therefore, we found that the expression of *kanC_opt_* or *tobC_opt_* improved the DOI titer as compared with *btrC*, which has been in common use according to the existing publications (Kogure et al., 2007; Tokuda et al., 2011; Watanabe et al., 2018). Thus, it was suggested that the expression of DOI synthases (orthologous to different species) that are involved in the same catalytic reaction could yield diverse DOI titers, whereas it was found that an improved DOI titer, as compared to the results from preceding studies (Kogure et al., 2007; Tokuda et al., 2011), could be achieved through the expression of artificially synthesized genes based on synthetic biology that takes into account codon usage preferences of the recombinant strains.

**Table 1:** 2-Deoxy-*scyllo*-inosose titers shown by the recombinant *B. subtilis* strains during fed-batch fermentation.

Strain	Genotype specification	DOI (g/L)
BSDOI-01	*Bacillus subtilis* 168 [BS] + pHC13-P_43_	Not detectable
BSDOI-02	BS + pHC13- P_43_-*btrC*	2.3 ± 0.2
BSDOI-03	BS + pHC13- P_43_-*genC_opt_*	0.8 ± 0.2
BSDOI-04	BS + pHC13- P_43_-*kanC_opt_*	3.4 ± 0.3
BSDOI-05	BS + pHC13- P_43_-*tobC_opt_*	3.6 ± 0.2
BSDOI-06	BSΔpgi	Not detectable
BSDOI-07	BSΔpgi + pHC13- P_43_-*btrC*	16.7 ± 0.7
BSDOI-08	BSΔpgi + pHC13- P_43_-*genC_opt_*	5.5 ± 0.7
BSDOI-09	BSΔpgi + pHC13- P_43_-*kanC_opt_*	22.5 ± 2.0
BSDOI-10	BSΔpgi + pHC13- P_43_-*tobC_opt_*	24.2 ± 1.4
BSDOI-11	BSΔpgiΔpgcA	Not detectable
BSDOI-12	BSΔpgiΔpgcA + pHC13- P_43_-*btrC*	20.7 ± 1.1
BSDOI-13	BSΔpgiΔpgcA + pHC13- P_43_-*genC_opt_*	6.4 ± 1.0
BSDOI-14	BSΔpgiΔpgcA + pHC13- P_43_-*kanC_opt_*	29.0 ± 2.9
BSDOI-15	BSΔpgiΔpgcA + pHC13- P_43_-*tobC_opt_*	37.2 ± 2.4


### Comparison of DOI Titers Produced by the Metabolically Engineered Recombinant Strains That Express *btrC*

DOI productivity of *btrC*-expressing recombinant strains (BSDOI-07 and BSDOI-12), in which either one or both *pgi* (Glc6P isomerase gene) and *pgcA* (phosphoglucomutase gene) involved in the glycolysis metabolic pathway was (or were) disrupted in the genome of *B. subtilis* as a host, was compared with that of the *btrC*-expressing BSDOI-02. BSDOI-07 represents the *btrC*-expressing Δpgi strain, whereas BSDOI-12 denotes the *btrC*-expressing ΔpgiΔpgcA strain of *B. subtilis*. The resulting strains BSDOI-07 and BSDOI-12 showed average DOI productivity of 16.7 and 20.7 g/L, respectively, which was more than seven- and ninefold higher than the DOI productivity (2.3 g/L) of the control (BSDOI-02), which did not undergo a metabolic pathway modification (**Table 1**). In other words, when two kinds of enzymes (such as Glc6P isomerase and phosphoglucomutase that utilize Glc6P as the common substrate within the branched glycolytic metabolic pathway) were deleted in the host, intracellular accumulation of Glc6P as a typical substrate of DOI synthase was induced, thus leading to significantly elevated DOI productivity through catalysis by the BtrC enzyme. Moreover, the aforementioned three kinds of recombinant strains did not show noticeable differences in cell growth during the shake-flask fermentation process.

### Comparison of DOI Titers Among the Metabolically Engineered Recombinant Strains That Express Codon-Optimized Artificial Genes

A natural *btrC* gene introduced into the Δpgi strain (BSDOI-7) as described above was replaced by three different kinds of codon-optimized artificial genes such as *genC_opt_, kanC_opt_*, and *tobC_opt_*, yielding recombinant strains BSDOI-08 to BSDOI-10. Moreover, a similar gene replacement process was carried out in the ΔpgiΔpgcA strain (BSDOI-12), thereby generating recombinant strains BSDOI-11 to BSDOI-13. The DOI titer produced by the recombinants was compared with that of two recombinant strains: BSDOI-07 and BSDOI-12. At first, among the recombinant strains with single deletion of the *pgi* gene, additional expression of artificial gene *genC_opt_* showed a noticeably decreased DOI titer (5.5 g/L) relative to the *btrC*-expressing BSDOI-07 strain (**Table 1**). Nonetheless, replacement of *btrC* with other codon-optimized DOI synthases (*kanC_opt_* and *tobC_opt_*) showed an improved DOI titer (average of 22.5 and 24.2 g/L, respectively) as compared to the control (average of 16.7 g/L). Similarly, recombinant strains with double deletion of genes *pgi* and *pgcA* showed a similar pattern of DOI titer; in particular, expression of artificial gene *tobC_opt_* in the above-mentioned recombinant host further increased DOI production to 37.2 g/L, on average (**Table 1** and **Figure 2**). Judging by the results above, engineering the glycolytic metabolic pathway in the host enables intracellular accumulation of Glc6P, and furthermore, the highest DOI titer and productivity were accomplished by means of artificial genes, as compared to other studies (Kakinuma et al., 2000; Kogure et al., 2007; Tokuda et al., 2011) that employed enzymatic reactions or an engineered recombinant strain of *E. coli*. Therefore, by combining the synthetic biology approach that spurs DOI biosynthesis in a metabolically engineered heterologous host *E. coli* strain, a maximum DOI production of 37.2 g/L was achieved. Hence these results highlight the above-mentioned 16-fold higher DOI titer compared with that of the *btrC*-expressing recombinant.

### Fed-Batch Fermentation by the BSDOI-15 Recombinant Strain

**FIGURE 3:**
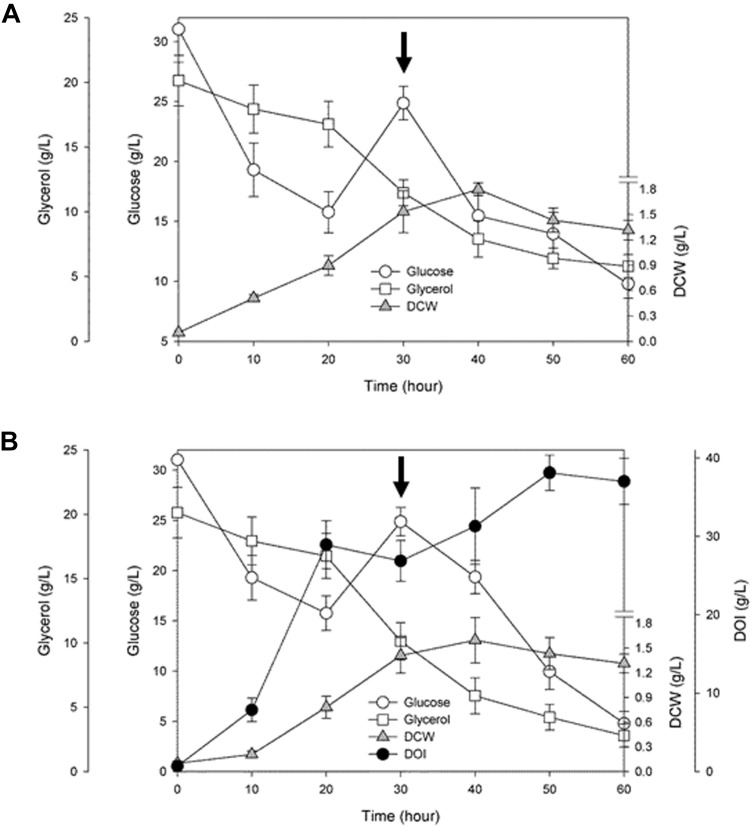
Time courses of cell growth and 2-deoxy-*scyllo*-inosose (DOI) production, together with the profiles of glucose and glycerol consumed during fed-batch fermentation by the recombinant **(A)** BSDOI-11 (BSΔpgiΔpgcA), and **(B)** BSDOI-15 (BSΔpgiΔpgcA + *tobCopt*) strain. DCW represents dry cell weight, whereas the arrow shown at 30 h denotes when 10 g of glucose was added. Data were expressed as median (*n* = 4) ± standard deviations.

To examine time courses of cell growth and DOI production during the fermentation driven by a resultant engineered BSDOI-15 cell factory, the culture broth was collected every 10 h. The dry cell weight and the fermentation profiles of glucose and glycerol, together with the DOI titer were quantitatively determined (**Figure 3** and **Supplementary Figures 1, 2**). Bacterial cell growth showed a typical fed-batch culture pattern, indicating that besides glucose, glycerol can serve as an extra carbon source for the growth of these DOI-producing cell factories. In fed-batch fermentation by the BSDOI-15 recombinant where glucose was added after 30 h fermentation, the profile of DOI production appears to closely correlate with that of glucose consumed. However, by the first 20 h of fed-batch fermentation, the concentration of glucose consumed was about 17.6 g/L, whereas DOI production was up to 27.9 g/L. Ideally, there should be one to one correspondence between the both concentrations. These discrepancies in reciprocal correlations between the glucose consumption and DOI production within 20 h fermentation might be due to the usage of complex media composition yeast extract. Further studies using chemically define medium, instead complex medium, could be a clue to the above question. Meanwhile, contrary to the sharp glucose consumption rate during the initial 20 h fermentation, the consumption rate of glycerol appears to be gradual slope; the intactness of Zwf on the pentose phosphate pathway during fed-batch fermentation will make extra flux to glyceraldehyde-3-P along with its precursors, thus causing catabolite repression of glycerol. A maximum DOI production of 38.0 g/L was reached at 50 h of fermentation, and then the DOI titer slightly decreased up to hour 60. Cell growth also seems to be in the stationary phase after 50 h fermentation, thus suggesting that there may be some relations between DOI production and cell stability in fed-batch fermentation. During the total 60 h of fermentation, the initial glucose level was set to 30 g per liter of a culture medium. Considering that 10 g was additionally fed into the culture after 30 h fermentation, a total of 40 g of glucose as the main carbon source was converted to 38.0 g of DOI, meaning that a yield of approximately 95% was achieved. The yield of 95% seen in the present study is lower than the 99% yield achieved in a metabolically engineered recombinant strain of *E. coli* into which a natural DOI synthase–encoding *btrC* gene was introduced (Kogure et al., 2007), but the DOI titer was higher than what was reported in this previous publication (i.e., 38.0 vs. 29.5 g/L). Furthermore, considering the DOI productivity based on total fermentation time (i.e., 50 vs. 72 h), the DOI productivity obtained in this study was 0.76 g/(L.h) compared with 0.41 g/(L.h) in the previous study on the engineered *E. coli* recombinant. Our result represents ∼1.8-fold enhancement of DOI productivity. Meanwhile, when the titers obtained from shake-flask and fed-batch fermentations were compared, there was no significant difference (i.e., 37.2 vs. 38.0 g/L). But, considering with the time-dependent productivity (i.e., 60-h vs. 50-h), the DOI productivity during shake-flask fermentation [0.62 g/(L.h)] was meaningfully lower than fed-batch fermentation [0.76 g/(L.h)]. Consequently, it was confirmed that the expression of a codon-optimized DOI synthase–encoding *tobC_opt_* gene in a metabolically engineered cell factory of *B. subtilis*—in which both the Glc6P isomerase *pgi* gene and phosphoglucomutase *pgcA* gene (involved in the glycolytic metabolic pathway) are disrupted—led to an approximately 38 g/L DOI titer within 50 h of fermentation that employs glycerol (besides glucose) as an extra carbon and energy source for growth. Namely, mass production of the desired DOI could be attained via the fed-batch fermentation by the engineered cell factory constructed through convergence of metabolic engineering and synthetic biology.

## Conclusion

Here, we report that the titer, yield, and productivity of DOI obtained via fed-batch fermentation by newly engineered *B. subtilis* cell factories are at least equivalent to those in another study [on engineered *E. coli* recombinants] (Kogure et al., 2007). For the production of DOI via fermentation by the recombinants constructed herein, we employed a dual carbon source: glucose and glycerol. Besides, in the case of the above-mentioned study on engineered *E. coli* recombinants, glucose and mannitol (up to 4%, fed into the fermentation medium) were utilized for DOI production. Glycerol has been generated as the main byproduct of the manufacture of biodiesel, one of renewable energy sources for the substitution of petroleum, thus pointing to the potential utilization of glycerol as a carbon or energy source for industrial fermentation (da Silva et al., 2009). Hence, in comparison with cost-ineffective mannitol, glycerol is surely a favorable and cost-effective carbon source for industrial-scale fermentation. Although further optimization of the fermentation parameters (i.e., the ratio of carbon sources or the cell mass or density corresponding to the modification of carbon sources, and the time point of feeding) is required, it will be useful to determine whether other cost-effective and sustainable carbon sources can be utilized to reach an equally high titer or productivity by means of these engineered recombinants. In this study, for the expression of natural and artificial DOI synthase-encoding genes, a strong constitutive P_43_ promoter was employed as the control. However, the recent studies on promoter screening or its engineering for fine-tuned gene expression in *B. subtilis* host gave us a more details on the host expression patterns (Song et al., 2016; Liu et al., 2018), which are essential for synthetic biology and metabolic engineering approaches. Development of other inducible promoters and their application onto our host could be useful for industrial production of DOI. In addition, owing to the slight drop of the DOI titer after 50 h of fermentation, monitoring of DOI profiles during the fed-batch fermentation may be necessary for the maintenance of DOI stability. In particular, DOI synthase acts on Glc6P to biosynthesize DOI with the help of cofactor nicotinamide adenine dinucleotide (NAD) (Huang et al., 2005; Nango et al., 2008). Redox engineering has often been carried out and applied to various desired products (Qiao et al., 2017). Therefore, as a further study, redox engineering, or subsequent engineering of the NAD recycling (or regeneration) pathway in the *B. subtilis* recombinants constructed in this study, may be tested as a potential booster not only to enhance the DOI titer or productivity but also to develop cell factory platforms relevant to DOI-based pharmaceuticals and fine chemicals.

## Author Contributions

JP and YY conceived the project and wrote the manuscript. JHL, HH, NL, JWL, ES, HS, and HK designed and conducted all the experiments. JHL, HH, NL, YY, and JP analyzed the results.

## Conflict of Interest Statement

The authors declare that the research was conducted in the absence of any commercial or financial relationships that could be construed as a potential conflict of interest.

## Abbreviations

DOI: 2-Deoxy-*scyllo*-inosose
Glc6P: glucose-6-phosphate
