# Supplementary material for: Enhanced Biosynthesis of 2-Deoxy-scyllo-inosose in Metabolically Engineered Bacillus subtilis Recombinants
Source: Front Microbiol. 2018 Sep 27;9:2333. doi: 10.3389/fmicb.2018.02333 (PMC6170601; doi:10.3389/fmicb.2018.02333)
Supplement: Supplementary file 1 [file Data_Sheet_1.PDF]

SUPPLEMENTARY

Enhanced Biosynthesis of 2-Deoxy-*scyllo*-inosose in Metabolically Engineered *Bacillus subtilis* Recombinants

Joo Hyun Lim[1,4], Hyun Ha Hwang[1,4], Na Joon Lee[1,4], Jae Woo Lee[1], Eun Gyo Seo[1], Hye Bin Son[1], Hye Ji Kim[1], Yeo Joon Yoon[3] and Je Won Park[1,2]*

[1]Department of Integrated Biomedical and Life Sciences, Graduate School, Korea University, Seoul, Republic of Korea
[2]School of Biosystem and Biomedical Science, Korea University, Seoul, Republic of Korea
[3]Department of Chemistry and Nanoscience, Ewha Womans University, Seoul, Republic of Korea
[4]These authors contributed equally to this work.

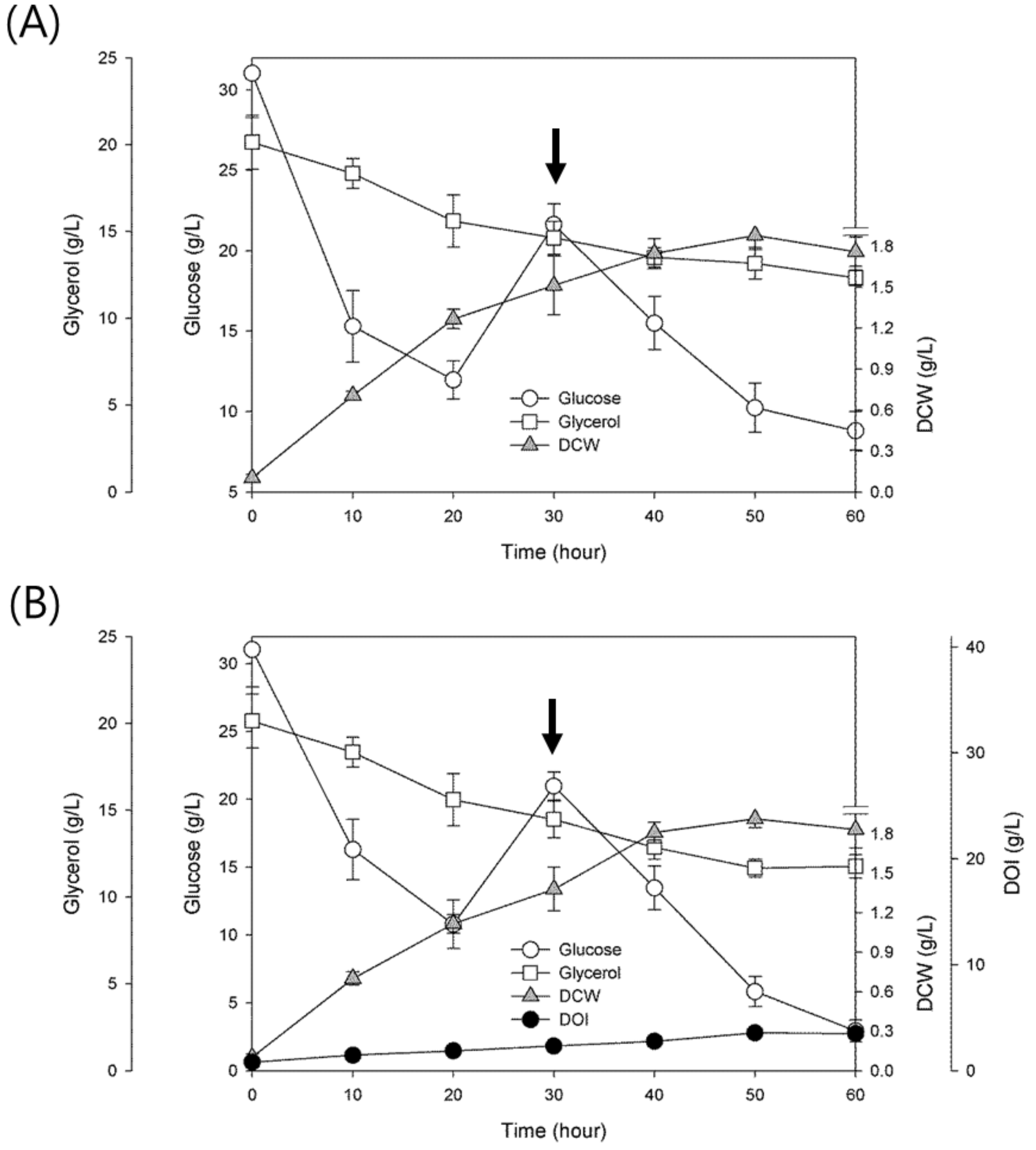

Supplementary Figure 1. Time courses of cell growth and 2-deoxy-*scyllo*-inosose (DOI) production, together with the profiles of glucose and glycerol consumed during fed-batch fermentation by the recombinant (A) BSDOI-01 (BS168), and (B) BSDOI-05 (BS168 +*tobCopt*) strain. DCW represents dry cell weight, whereas the arrow shown at 30 h denotes when 10 g of glucose was added. Data were expressed as median (n=4) ± standard deviations.

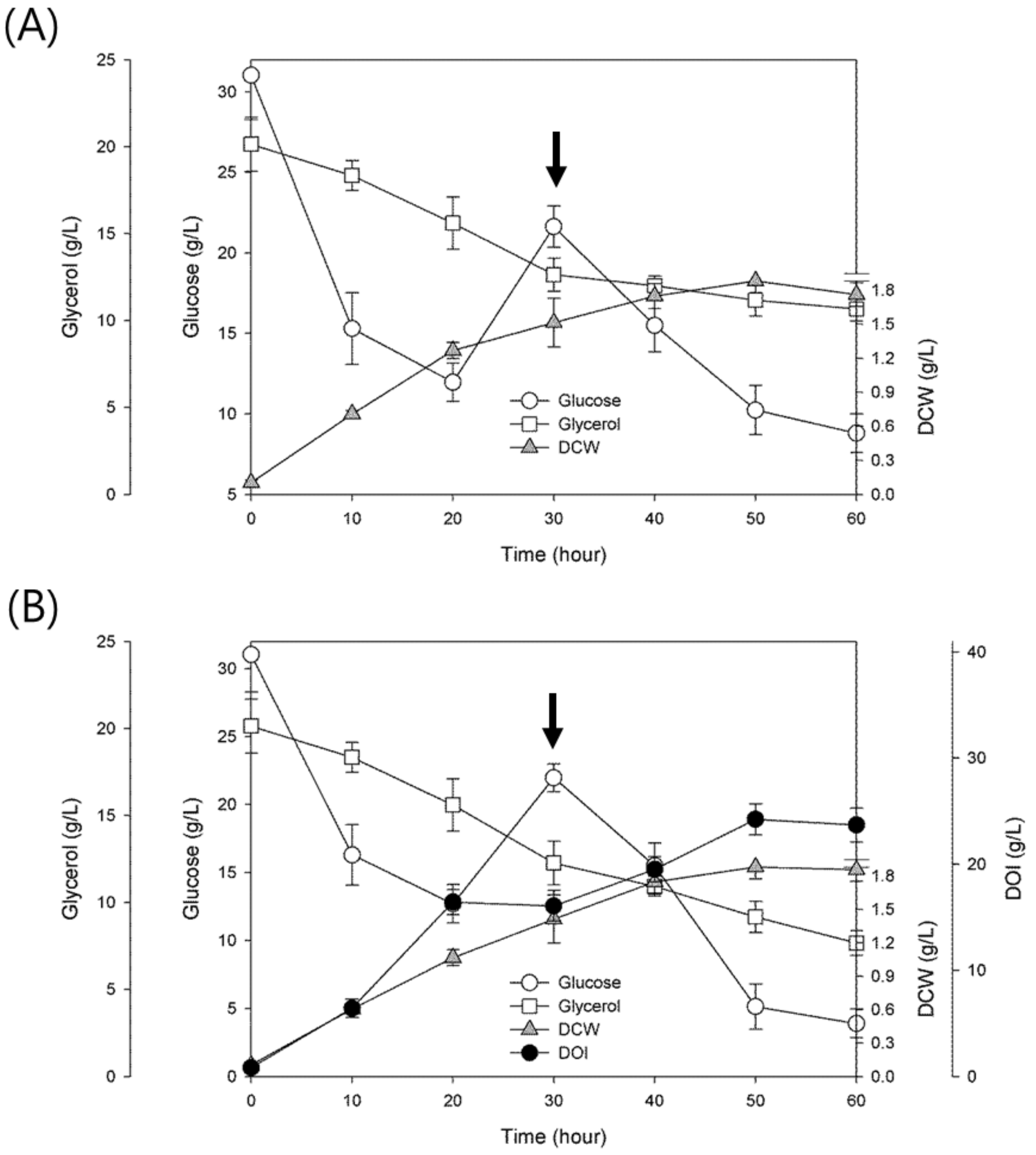

Supplementary Figure 2. Time courses of cell growth and 2-deoxy-*scyllo*-inosose (DOI) production, together with the profiles of glucose and glycerol consumed during fed-batch fermentation by the recombinant (A) BSDOI-06 (BSΔpgi), and (B) BSDOI-10 (BSΔpgi +*tobCopt*) strain. DCW represents dry cell weight, whereas the arrow shown at 30 h denotes when 10 g of glucose was added. Data were expressed as median (*n*=4) ± standard deviations.
